# A retrospective analysis of leucocyte count as a strong predictor of survival for patients with acute paraquat poisoning

**DOI:** 10.1371/journal.pone.0201200

**Published:** 2018-07-25

**Authors:** ShunYi Feng, Jie Gao, Yong Li

**Affiliations:** Emergency Department, Cangzhou Central Hospital, Yunhe Qu, Cangzhou City, China; Università degli Studi di Milano, ITALY

## Abstract

The aim of this study is the identification of a reliable predictor of prognosis to optimize the treatment of acute paraquat (PQ) poisoning patients. We performed a retrospective analysis on 96 patients with acute PQ poisoning to evaluate leucocyte count as a predictor of 90-day survival. These patients were admitted to the emergency department from May 2012 to February 2017. Kaplan–Meier method was used to compare the 90-day survival. Cox proportional hazard models were utilized to estimate the hazard ratios (HR) and 95% confidence intervals (CI). Receiver operating characteristic (ROC) analysis was conducted to analyze the discriminatory potential of leucocyte with respect to 90-day survival. Result showed that leucocyte was significantly higher among nonsurvivors than that among survivors (p<0.001). Leukocyte was also an independent predictor of survival according to the multivariate Cox analysis (HR 1.103; 95%CI: 1.062–1.146; p<0.001). The area under the curve (AUC) for leucocyte (AUC 0.911; 95%CI: 0.855–0.966; p<0.001) showed a discriminatory potential similar to that of the plasma PQ concentration (AUC 0.961; 95%CI: 0.926–0.997; p<0.001) in predicting 90-day survival. The leucocyte count is a strong predictor of survival in patients with acute PQ poisoning.

## Introduction

Paraquat (*N*,*N*′-dimethyl–4,4′–bipyridinium dichloride; PQ) is a widely used effective herbicide with favorable environmental characteristics and cost effectiveness. PQ poisoning exhibits low survival rate, especially in patients with moderate to severe poisoning; this poisoning may result in acute renal failure, hepatitis, and pulmonary fibrosis, which often lead to death within several weeks. Intentional self-poisoning with PQ is an important public health problem in the Asia–Pacific region with an estimated 300,000 deaths annually [1].

A reliable predictor of prognosis is useful for the future treatment of acute PQ poisoning patients. For example, the early prediction of prognosis could allow a more suitable therapy for patients having the best predictable survival rate and stimulate new research for the treatment of patients, who have the intermediate or worst prognostic parameters [2]. To date, no prognostic models have been prospectively validated because of issues in their development, such as small sample sizes, differences in the degree of severity, and complicated exclusion criteria [3]. Currently, the most remarkable potential prognostic marker that can predict survival is the plasma PQ concentration with acceptable sensitivity and specificity [2, 4] However, plasma PQ concentration measurement is unavailable in many hospitals.

Some studies indicated the increased leucocyte levels in patients with acute PQ poisoning [5, 6]. Nevertheless, the relationship between initial leucocyte level and survival has not yet been investigated, despite that the methods used to assess leucocyte levels are easily performed and relatively inexpensive. To determine whether the abnormal leucocyte is a good predictor of survival in patients with acute PQ poisoning, we performed a retrospective study to analyze patients with acute PQ poisoning. These patients were admitted to the emergency ward from May 2012 to February 2017.

## Material and method

This retrospective clinical study was conducted in accordance with the Declaration of Helsinki and approved by the Medical Ethics. The Institutional Review Board approval was obtained, but not the specific informed consent from patients due to the retrospective review of existing data. However, written consents regarding the risks associated with acute PQ poisoning and all treatment modalities (particularly charcoal hemoperfusion, glucocorticoid, and cyclophosphamide) were obtained from all patients upon their initial admission. Patient records and information were anonymized.

### Patients

A retrospective study was conducted in 96 patients with acute PQ poisoning (38 males and 58 females) with a mean age of 39 years. These patients were treated between May 2012 and February 2017 at the Emergency Department. Patients were diagnosed with acute PQ poisoning based on initial symptom presentation or self-reported exposure. The diagnosis was subsequently confirmed with plasma PQ testing. Upon arrival at the emergency room, blood samples were obtained from patients to determine the PQ concentration.

### Protocol for paraquat detoxification

A unified therapeutic regimen, including gastric lavage, catharsis, fluid replacement, diuresis, antioxidants (Vitamin C, Vitamin B, and L-Glutathione), immunosuppressant (corticosteroid), hemoperfusion, and organ-support therapy, was given to all the patients.

### Inclusion and exclusion criteria

The inclusion criteria were as follows: (1) confirmed diagnosis of acute PQ poisoning by plasma PQ testing, (2) aged >14, (3) presented PQ poisoning by oral intake, and (4) arrived at the Emergency Department within 8 h after ingestion. Exclusion criteria were as follows: (1) dermal or intravascular exposure; (2) with history of severe heart, lung, liver, kidney, or hematological system diseases; (3) with multiple organ failure; (4) pregnant or lactating; (5) with infection; or (6) with cancer.

### Data collection

Data provided by the patients were collected by four well-trained physicians using a standard data collection form. Blood samples for the initial clinical parameters, including leucocyte count, alveolar oxygen partial pressure (PaO_2_), creatinine, alanine aminotransferase (ALT), and plasma PQ concentration (tested by HPLC), were obtained. Survival time was determined from medical records or telephone follow-up. The survivors were described as patients who survived for 90 days after PQ ingestion. The normal plasma leucocyte level was set at 3.5–10×10^9^/L.

### Statistical analysis

All analyses were conducted using the SPSS version 13 (SPSS Inc., Chicago, IL, USA). Differences with a p-value of <0.05 were considered statistically significant. Nominal data are presented as frequencies and percentages, and continuous variables are presented as mean and standard deviation or median and interquartile range after assessments for normality of distribution. The chi-square test or Fisher’s exact test was used for comparisons of nominal variables. The sample *t*-test and Mann–Whitney U-test were utilized to compare continuous variables. To assess the relationship between leucocyte level and survival, the Kaplan–Meier survival curves were compared with the log-rank test. A univariate Cox regression analysis was performed to compare the frequency of potential risk factors associated with 90-day survival. Similarly, to control the confounding factors, a multivariable Cox regression analysis was carried out to analyze the factors that were significant on univariate analysis and satisfying the assumptions of a proportional hazard model. The criterion for significance to reject the null hypothesis was a 95% confidence interval (CI). Receiver operating characteristic (ROC) curves were computed, and areas under the curves (AUCs) were used to evaluate how well a model distinguishes the survivor group from the nonsurvivor group. Pairwise comparison of ROC curves between the areas for leucocyte and plasma PQ concentration was conducted by MedCalc using the method of Hanley and McNeil. Sensitivity, specificity, and cutoff value (sensitivity+specificity– 1) of various predictors/prediction models were also calculated to provide a complete description of the prediction parameters.

## Results

### Patient characteristics

**Fig 1:**
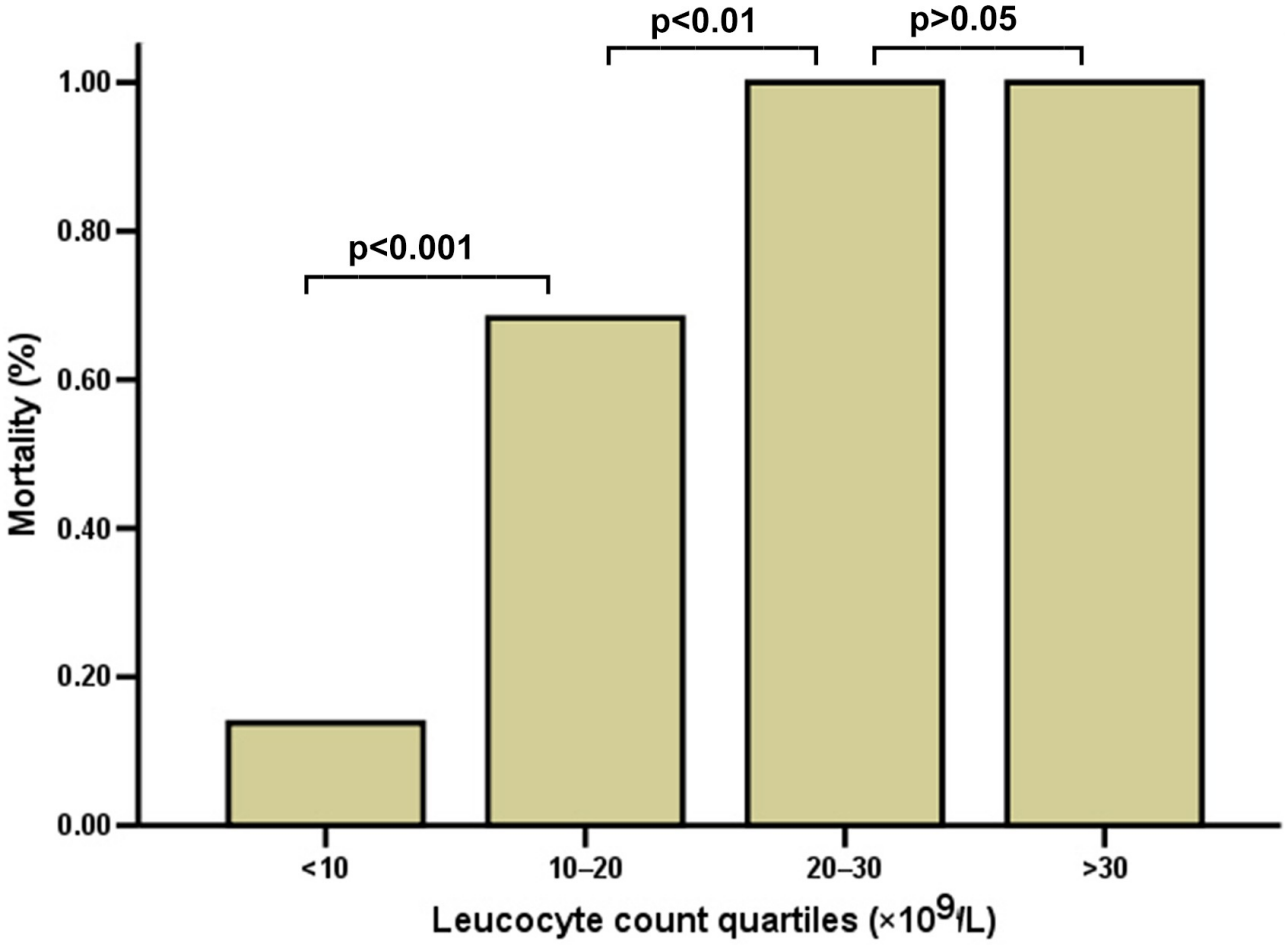
Mortality of the groups according to the leucocyte count quartile.

Among the 104 patients, 96 patients were enrolled in the study for further analysis, 5 patients presented incomplete data, 2 patients were not followed up, and 1 patient was transferred to another hospital. Within the 90-day follow-up period after poisoning, 58 patients succumbed to poisoning, and 38 patients survived, with a survival rate of 39.6%. As shown in Table 1, leucocyte, ALT, creatinine, PaO_2_ at room air, and plasma PQ concentration upon arrival were all significantly higher in the mortality group than those in the survival group. Table 2 shows that ALT, creatinine, and plasma PQ concentration upon arrival were significantly different among groups stratified according to leucocyte level quartile. In addition, the mortaltiy rates were 14% in leucocyte <10×10^9^/L, 68% in leucocyte of 20×10^9^/L–30×10^9^/L, 100% in leucocyte of 20×10^9^/L–30×10^9^/L, and 100% in leucocyte >30×10^9^/L (Fig 1).

**Table 1:** General characteristics upon arrival between survival and mortality groups.

	Mortality group (*n* = 58)	Survival group (*n* = 38)	*P*
**Age (years)**	39.00 (32.00)	36.50 (21.25)	0.251
**Gender (male/female)**	22/36	16/22	0.683
**Time from ingestion to gastric lavage (h)**	1.00 (1.00)	1.00 (1.13)	0.288
**Leucocyte (×10**^**9**^**/L)**	19.22 (10.87)	8.78 (5.05)	<0.001
**Alanine aminotransferase (ALT, μ/L)**	32.85 (16.33)	26.80 (9.30)	0.009
**Creatinine (umol/L)**	104.50 (73.50)	62.50 (20.50)	<0.001
**Alveolar oxygen partial pressure (PaO**_**2,**_ **mmHg) **	89.49±9.95	94.13±9.25	0.024
**Plasma paraquat (PQ) concentration (ng/mL)**	3.85 (9.00)	0.35 (0.83)	<0.001

**Table 2:** General characteristics upon arrival stratified according to leucocyte count quartiles.

	<10×10^9^/L (*n* = 29)	10–20×10^9^/L (*n* = 41)	20–30×10^9^/L (*n* = 21)	>30×10^9^/L (*n* = 5)	*P*
**Gender (male/female)**	13/16	15/26	8/13	2/3	0.971
**Time from ingestion to gastric lavage (h)**	1.00 (0.50)	1.00 (1.00)	1.00 (1.75)	2.00 (1.70)	0.201
**Alanine aminotransferase (ALT, μ/L)**	27.00 (9.30)	28.40 (15.20)	32.50 (15.85)	38.00 (27.50)	0.007
**Creatinine (umol/L)**	66.00 (24.50)	73.00 (40.50)	141.00 (77.00)	154.00 (72.50)	<0.001
**Alveolar oxygen partial pressure (PaO**_**2,**_ **mmHg) **	93.98±10.38	91.38±8.06	88.60±12.47	86.94±5.44	0.200
**Plasma paraquat (PQ) concentration (ng/mL)**	0.20 (1.00)	2.10 (3.85)	5.00 (9.05)	20.30 (30.30)	<0.001

### Kaplan–Meier survival analysis and risk factor analysis for 90-day survival

**Fig 2:**
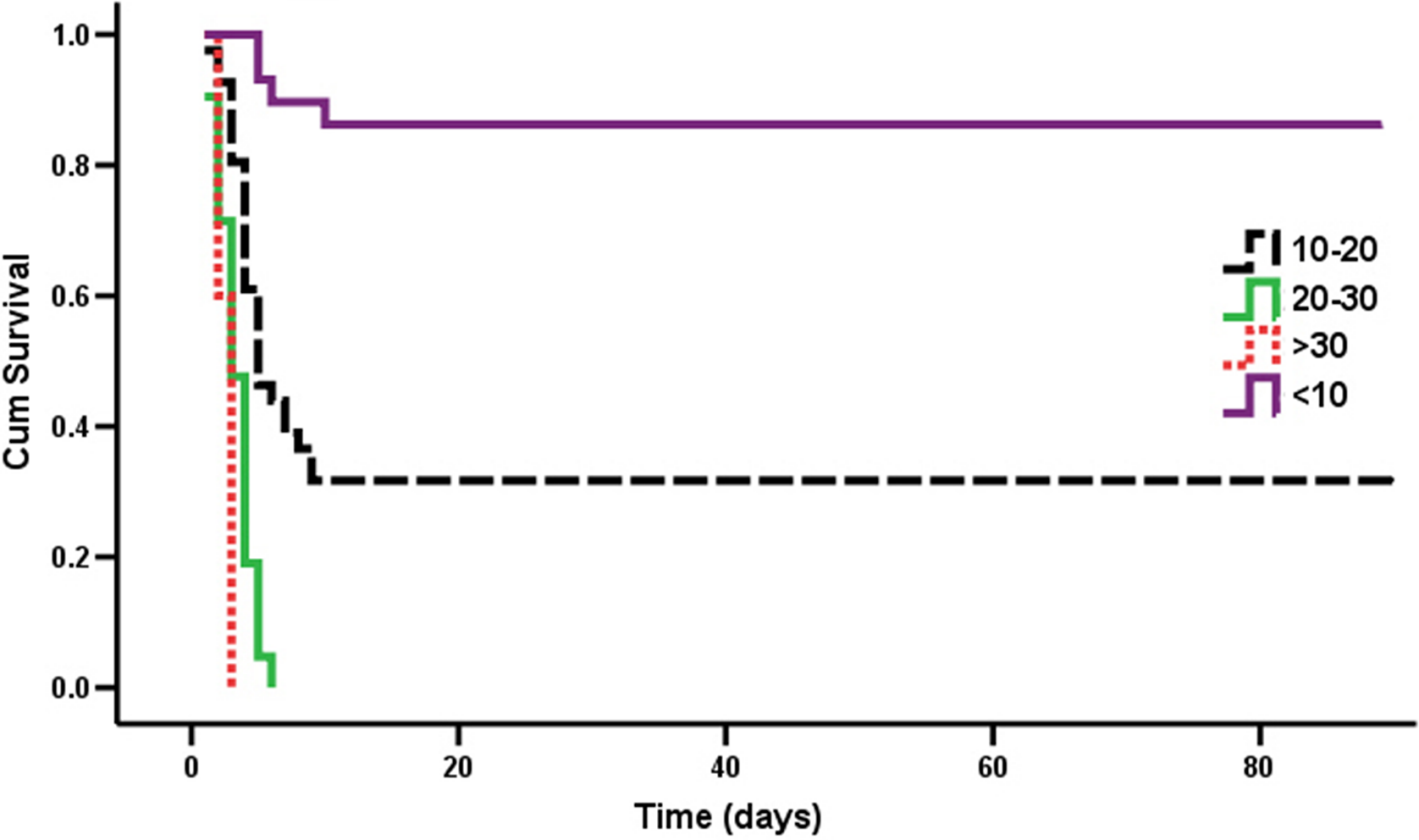
Kaplan–Meier analysis of survival curves for the groups according to the leucocyte count quartile.

A log-rank test confirmed that categorized leucocyte was associated with high 90-day survival (95% CI: 29.684–46.504; p<0.001) (Fig 2). In the univariate Cox proportional hazards regression analyses, leucocyte, ALT, plasma creatinine, PaO_2_ at room air, and plasma PQ concentration upon arrival were associated with the risk of 90-day survival from PQ poisoning. In the multivariate Cox proportional hazards regression analyses, only leucocyte, creatinine, and plasma PQ concentration upon arrival were independent prognostic factors. Moreover, patients with an increased leucocyte level of 10×10^9^/L or higher showed a higher risk of death than that of patients with normal leucocyte level during the 90-day follow-up period (Table 3).

**Table 3:** Cox regression model.

	Univariate COX model HR (95% CI)	*P*	Multivariate COX model HR (95% CI)	*P*
**Age (years)**	1.010 (0.995–1.024)	0.199	N/A	
**Gender (male/female)**	0.846 (0.497–1.438)	0.536	N/A	
**Time from ingestion to gastric lavage (h)**	0.969 (0.801–1.171)	0.774	N/A	
**Plasma paraquat (PQ) concentration (ng/mL)**	1.071 (1.050–1.092)	<0.001	1.028 (1.005–1.052)	0.017
**Alanine aminotransferase (ALT, μ/L)**	1.020 (1.003–1.037)		N/A	
**Creatinine (umol/L)**	1.004 (1.002–1.005)		1.004 (1.002–1.006)	0.001
**Alveolar oxygen partial pressure (PaO**_**2,**_ **mmHg)**	0.971 (0.944–0.988)		N/A	
**Leucocyte (×10**^**9**^**/L)**	1.133 (1.096–1.171)		1.103 (1.062–1.146)	<0.001
** <10**	reference		reference	
** 10–20**	7.622 (2.660–21.837)	<0.001	6.564 (2.272–18.968)	<0.001
** 20–30**	24.712 (8.1109–75.309)	<0.001	14.757 (4.677–46.566)	0.001
** >30**	51.331 (12.527–210.342)	<0.001	12.983 (2.367–71.220)	0.001

N/A = not applicable

### ROC analysis

**Fig 3:**
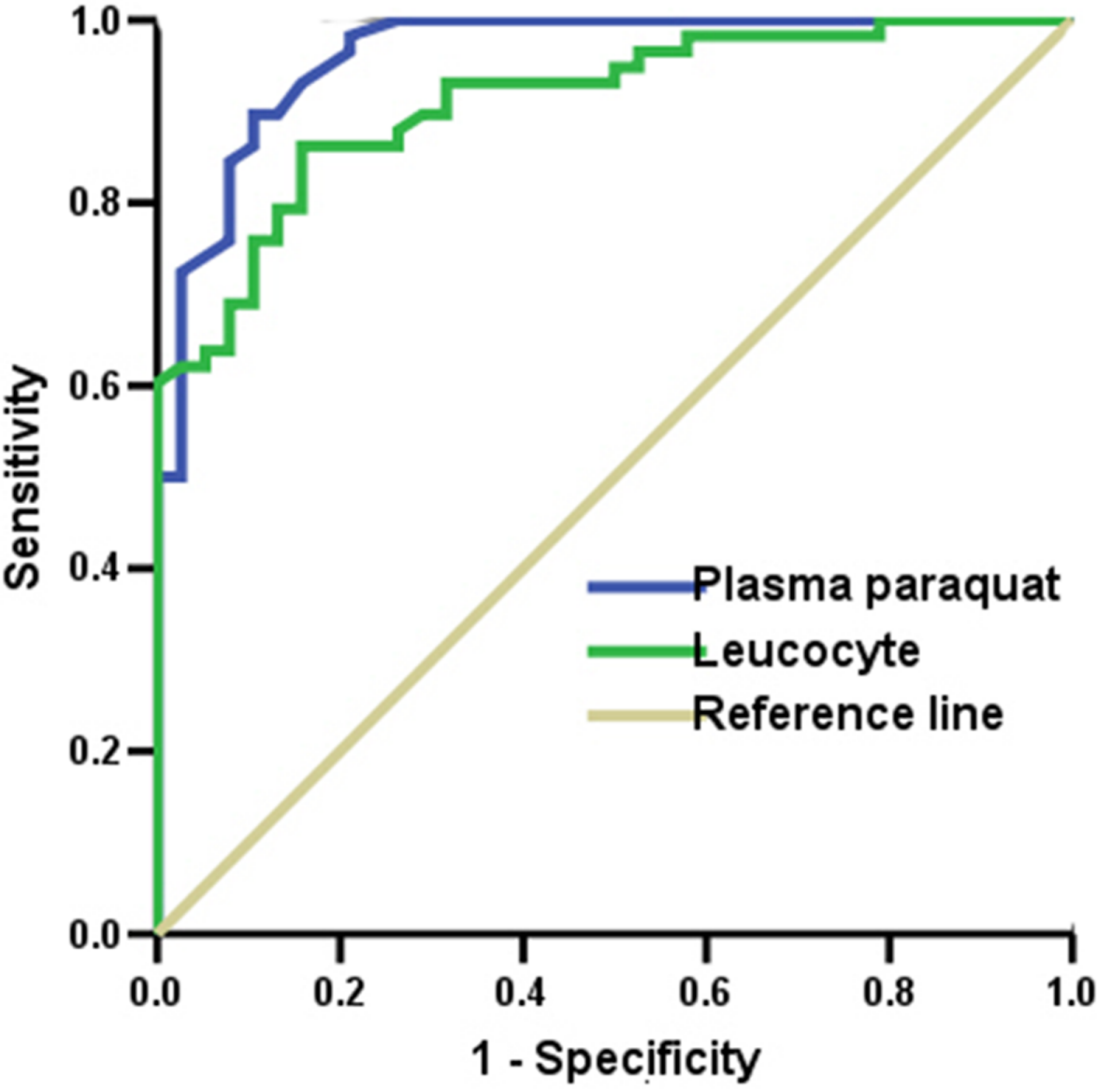
Area under the receiver operating characteristic curve analysis.

The ROC curve of leucocyte showed an AUC of 0.911 (95% CI: 0.855–0.966, p<0.001). The most remarkable cut-off value for leucocyte was 11.78×10^9^/L, with a sensitivity of 86.2% and a specificity of 84.2% (Fig 3). The ROC curve of plasma PQ concentration presented an AUC of 0.961 (95% CI: 0.926–0.997, p<0.001). The most remarkable cut-off value for plasma PQ concentration was 1.55 ng/mL, with a sensitivity of 89.7% and a specificity of 89.5%. Pairwise comparison of the ROC curves showed that no statistically significant difference existed between the areas for leucocyte and plasma PQ concentration (z = 1.632; p = 0.103).

## Discussion

The clinical features of acute PQ intoxication are characterized with low survival, rapid progression, and frequent injury to the lung and kidney. The case survival is considerably low in all centers despite large variations in treatment [3, 7–9] and the survival rate varies between 10% and 50%; in cases of intentional self-poisoning with concentrated formulations, mortality approaches 100% [10]. The overall survival rate in the present study was 39.6%, which is consistent with the survival rate of 10%–50% in previous reports [3, 7–9].

Some prediction models based on plasma PQ concentration have been suggested previously [11, 12]. Patients whose plasma PQ concentrations do not exceed 2.0, 0.6, 0.3, 0.16, and 0.1 mg/L at 4, 6, 10, 16, and 24 h, respectively, are likely to survive [4]. Nevertheless, determining which patients will survive in a clinical setting is also difficult with the use of these numerical predictive levels because some patients with low PQ concentration still die. Recently, Gil et al. [13] reported that the minimum PQ concentration of deaths is remarkably low (0.12, 0.02, and 0.01 **μ**g/mL at 5, 12, and 24 h, respectively), which indicated that plasma PQ concentration is not a good predictor of mortality in the low plasma PQ concentration. The outcome may be explained by pharmacokinetics of PQ. Some studies [14, 15] reported that the peak time of plasma PQ concentration is 1–3 h, and the peak time of lung cells is at 4–5 h. Within 5–6 h after ingestion, 90% of PQ disappears in the plasma. During this period, plasma concentration shows substantial variation with slight changes at different time intervals after ingestion. Furthermore, any uncertainty on ingestion time can result in uncertainty on survival estimates. Under clinical situation, plasma PQ concentration within 12 h may be reliable, but the curve in the nomogram is not discriminable thereafter; consequently, predicting the clinical outcome becomes difficult[16]. In the present study, the ROC curve of leucocyte displayed an AUC of 0.961, which is higher than that in previous report [17] because of short poisoning time (within 8 h) after ingestion in this study. In addition, Some toxicity signs, such as renal failure [18], high lactate [18], high APACHE II score[18], high lymphocyte count [5], peripheral burning sensation [19], changes on chest radiograph [20], gastrointestinal lesion [21], hypotension, severe hypoxia, acidosis, and low Glasgow Coma Scale [22] are used to differentiate those who are likely to succumb eventually from survivors. However, the use of sensory data may not provides highly reliable and accurate estimates of severity of their poisoning.

Previous studies demonstrated that leucocyte participates in the induction of organ system dysfunction in acute inflammatory processes, such as acute lung injury induced by PQ, where their capability to release ROS contributes to cellular and tissue damage [23]. The leucocyte count during admission is also emphasized as an index in predicting PQ poisoning outcomes [17, 24, 25]. These results suggest that abnormal leucocyte may provide as much information for the prediction of the prognosis as plasma PQ concentrations do. In our study, the median of leucocyte amount in the mortality group was 19.22×10^9^/L. This amount is more than two times of that in the survival group. In addition, the present study also presented that abnormal leucocyte level was negatively related to 90-day survival rate, that is, high leucocyte level indicates low survival rate. The leucocyte >20×10^9^/L within 8 h post-PQ ingestion indicates a survival rate of 0%. At 10×10^9^/L–20×10^9^/L, the survival rate still reached approximately 30%. Our results are similar to those in previous finding [5]. The lymphocyte, as a specific type of leucocyte, is categorized in quartiles, and the survival rates are 38.9% in lymphocyte <1700, 45.7% in 1700≤lymphocyte<3200, 28.8% in 3200≤lymphocyte<5000, and 0% in lymphocyte>5000. In addition, Zhou et al.[26] evaluated leucocyte count within the first 24 h of admission as a predictor of 30-day survival and demonstrated that leukocyte counts within the first 24 h of admission had an area of 0.849 (95%CI, 0.796–0.902). Thus, we speculate leukocyte count within the first 24 h is a valuable parameter in predicting the prognosis of PQ poisoning.

Our study encountered several limitations. First, no data were collected on the lymphocyte levels prior to PQ intoxication. Therefore, whether the increased leucocyte levels were entirely due to PQ ingestion remained unconfirmed. Second, the study was limited by its retrospective design and potential recall bias with an inability to recall the accurate death time. Third, the mechanism of the association between abnormal and patient leucocytes is unclear. Further studies should be conducted to investigate the exact mechanisms. Fourth, leucocyte count changes after the application of cyclophosphamide and corticosteroids, thereby resulting in uncertainty in discriminating between patients with no chance of survival and those who may potentially benefit from interventions in clinical trials using investigational antidotes.

## Conclusion

The abnormal leucocyte is a good predictor of survival in patients with acute PQ poisoning. Given that leukocyte count measurement is a common, low cost, and simple test, it may be useful for PQ risk assessment in individuals classified as low risk using traditional risk stratification tools. However, an ideal model that includes other factors, such as creatinine, amylase, and potassium, which make the prediction of survival/mortality scientific, rational, and quantitative, should be set up.

## Supporting information

S1 File STROBE_checklist_cohort. (DOC)
